# Fermat’s Principle of Least Time Predicts Refraction of Ant Trails at Substrate Borders

**DOI:** 10.1371/journal.pone.0059739

**Published:** 2013-03-20

**Authors:** Jan Oettler, Volker S. Schmid, Niko Zankl, Olivier Rey, Andreas Dress, Jürgen Heinze

**Affiliations:** 1 Biologie I, Universität Regensburg, Regensburg, Germany; 2 Centre de Biologie et de Gestion des Populations, Campus international de Baillarguet, Montferrier-sur-Lez cedex, France; 3 Chinese Academy of Sciences – Max-Planck-Gesellschaft Partner Institute for Computational Biology, Chinese Academy of Sciences, Shanghai, China; 4 infinity3 GmbH, Bielefeld, Germany; Arizona State University, United States of America

## Abstract

Fermat’s principle of least time states that light rays passing through different media follow the fastest (and not the most direct) path between two points, leading to refraction at medium borders. Humans intuitively employ this rule, e.g., when a lifeguard has to infer the fastest way to traverse both beach and water to reach a swimmer in need. Here, we tested whether foraging ants also follow Fermat’s principle when forced to travel on two surfaces that differentially affected the ants’ walking speed. Workers of the little fire ant, *Wasmannia auropunctata*, established “refracted” pheromone trails to a food source. These trails deviated from the most direct path, but were not different to paths predicted by Fermat’s principle. Our results demonstrate a new aspect of decentralized optimization and underline the versatility of the simple yet robust rules governing the self-organization of group-living animals.

## Introduction

The processes underlying biological decentralized organization have become models for modern-age human applications. In particular, the ability of ants to form efficient trails has been an inspiration to scientists solving complex problems in robotics, logistics and information technology [1]–[5]. In many ant species, workers traveling between the nest and food will deposit a trail of pheromones on the ground. Additional workers are recruited by these pheromones and the trail is reinforced through positive feedback [6], [7]. On homogeneous surfaces, trails will converge towards the most direct path between nest and food source [8], [9]. However, no study has examined how ants direct their trails on heterogeneous surfaces on which trail optimization is more complex. One way for ants to optimize their path across two surfaces would be to follow Fermat’s principle of least time, which posits that a ray of light traveling between two points follows the fastest (and not necessarily the most direct) route. At the boundary between two media in which light travels at different speeds, light refracts accordingly, thus minimizing travel time as a function of elementary physical properties. Unlike light, ants are under evolutionary selection and path formation results from the interaction of behavior, pheromone properties and the environment. Nevertheless, we used Fermat’s principle to predict the optimum for ant foraging, assuming (and simplifying) that ants are selected to minimize travel time. In particular, we tested whether ants foraging across two surfaces deviate from the most direct path but not from a time-optimized solution.

## Materials and Methods

### Experimental Set-up

**Figure 1:**
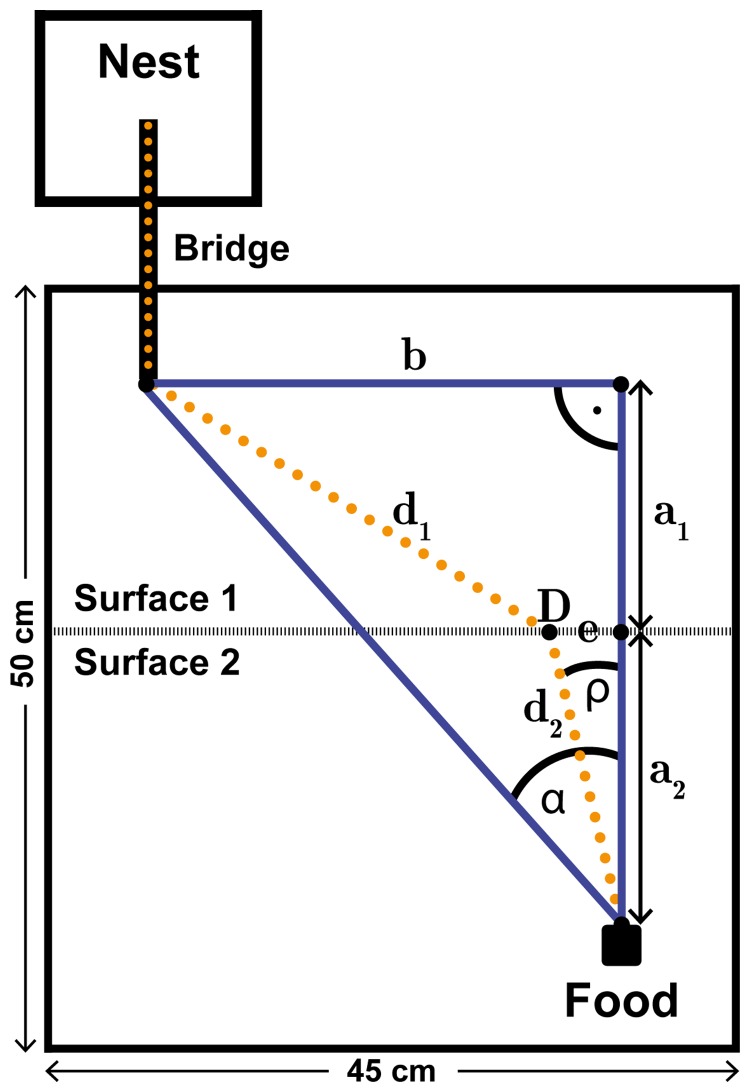
Schematic of the two-surface test illustrating the geometric details for deriving the path prediction model. The ants crossed two kinds of surfaces to gather food. The dotted yellow line indicates the trail expected according to Fermat’s principle. With decreasing walking speed ratio (*v*_1_/*v*_2_) point D (associated with the predicted trail angle *ρ*) will move to the left. If *v*_1_< *v*_2_, *ρ* will be larger than *α* (the crossing angle of the shortest line between food and nest).

We used colonies of the little fire ant *Wasmannia auropunctata*, one of the world’s 100 most invasive species [10]–[12]. Three colonies (each containing several thousand workers and multiple queens) were collected near the sites Herzliyya, Newe Yaraq and Ma’barot in Israel and kept in stacked plaster-floored petri dishes inside a plastic box. Similarly to most introduced and native *W. auropunctata* populations worldwide, the population in Israel is of unicolonial structure with clonal reproduction [13]. The plastic boxes were connected to one corner of a foraging arena by a cardboard bridge and cockroaches were provided as food *ad libitum* in the opposite corner. The surface of the foraging arena was split in halves, each half covered with one of three materials that differentially affected the ants’ walking speed (Fig. 1). We chose two types of polyester felt, rough (Rayher Hobby GmbH, Laupheim, Germany) and smooth (K.D.J. Brand GmbH&Co.KG, Offenbach, Germany), in addition to polyethylene (LDPE) glass (Caleppio/Charles Wolfsberger GmbH, Weil am Rhein, Germany).

**Figure 2:**
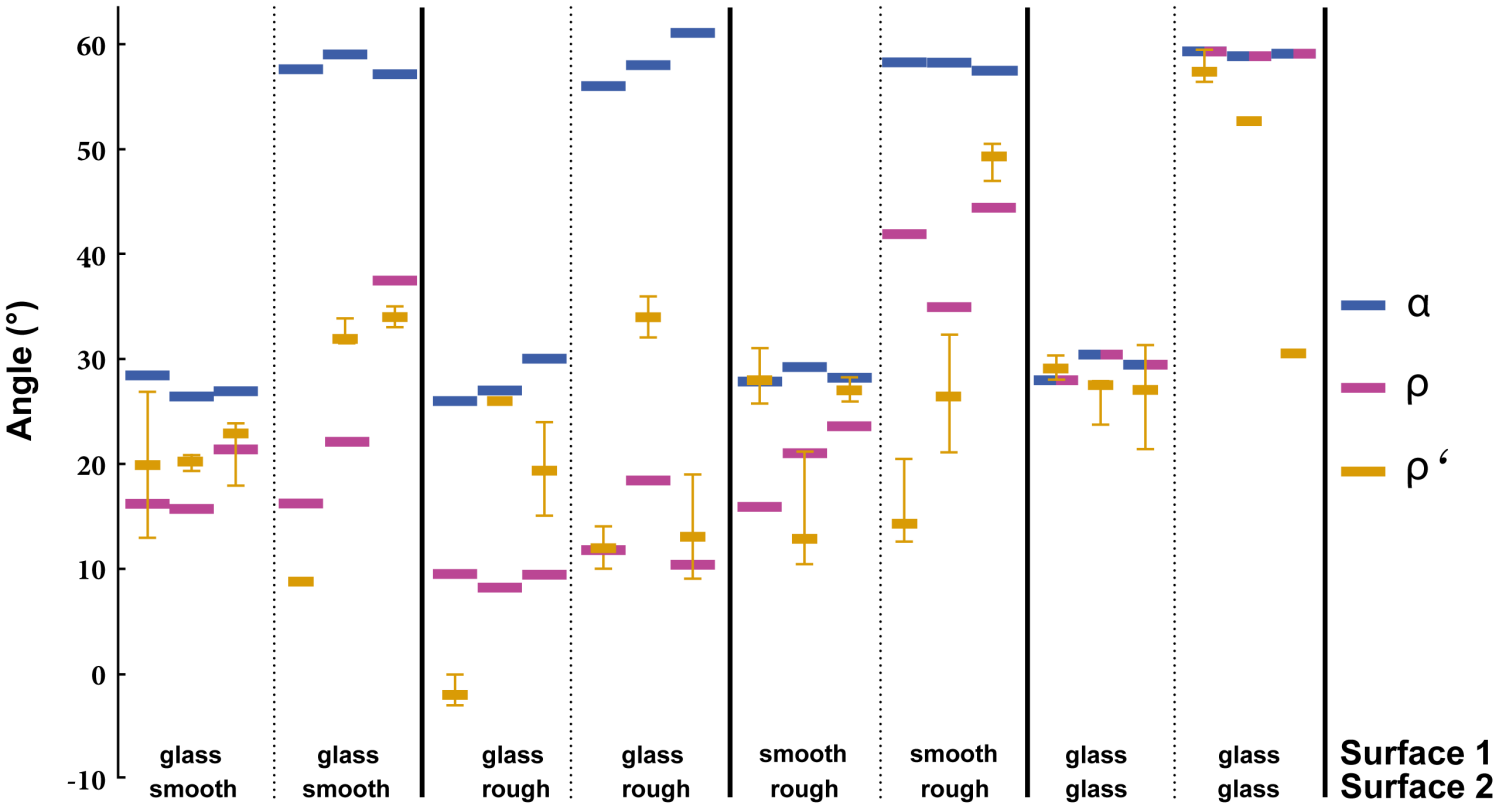
When forced to move across different surfaces, colonies of the ant *W. auropunctata* formed trails with an angle *ρ'* closer to the optimum *ρ* (in 15 out of 18 trials) than to the angle *α* they would have followed if moving to the food source on a straight line. Given for each surface combination are *α*, *ρ* and *ρ'* of each of three colonies (bars = median, whiskers = minimum and maximum). *ρ* varied largely due to colony differences in walking speed.

Given *α* (i.e., the angle defining the shortest path), the distances *a*_1_ and *a*_2_ and the walking speeds *ν*_1_ and *ν*_2_, we predicted the angle *ρ* at which ants would cross from one surface onto the second if they (eventually) chose the fastest path (Fig. 1). Control trials on a homogenous surface (glass – glass) demonstrated that, as expected, the ants chose a path close to that defined by *α* in five of six trials (see Fig. 2). We tested three surface combinations: Glass – smooth felt, glass – rough felt, smooth felt – rough felt. The food was offered at two different locations, resulting in large and small angles *α* (60° and 30°) for all combinations. Altogether, we conducted 18 test trials: three colonies on three surface combinations and two food positions each. (Henceforth, if not specified, “trials” refers to “test trials”.).

Each surface/geometry combination was tested for one week in parallel with each of the three colonies. After each trial, the bridges to the arenas were removed and the ants were returned into their nests. The next trial started after a two-day break, during which the ants had neither access to the arena nor to food. Each trial started without food, i.e. the foraging ants were allowed to explore the arena for one day before food was added. We conducted the trials in July 2010 (glass - smooth felt; glass - rough felt, both at *α = *30° and 60°, respectively) and in August 2011 (smooth felt - rough felt; glass - glass, both at *α = *30° and 60°, respectively). The last two trials were the controls.

Between the two series of trials the colonies were kept inside their nest boxes at room temperature and were fed *ad libitum*. The long interval between the two series makes it unlikely that the results of our experiments were affected by the previous experience of foragers.

Approximately two weeks prior to the trials in 2010 and 2011 the colonies were transferred to another room, kept at constant 25°C and in complete darkness (except when being tended, filmed or when experimental set-ups were changed) to prevent visual orientation and location of the food source.

### Geometry

The direction *ρ′* of the realized foraging trail was assessed every morning by taking a digital picture. Only data from the last three days of each trial were analyzed, assuming that trails required a few days to form a path close to the optimum. When a trail was diffuse, i.e. the ants were scattered around a straight line, we measured the two outermost edges and calculated the average. In two cases the trails were overly diffuse and we could only reliably use data from a single day, in both cases the last day. The angle *α* (denoting the shortest path) deviated slightly from 30° and 60° both within and among trials, because of minor variation in food position due to handling. The angle measurement was also subject to small optical distortions in the digital pictures. Thus, we daily measured the colony-specific angle *α* from those pictures the same way we measured *ρ′* and calculated the mean angle of *α* over the course of each trial (mean ± SD for n = 9 trials: *α_30_* = 28.79° ±1.09; *α_60_* = 58.57° ±0.71).

### Speed

**Figure 3:**
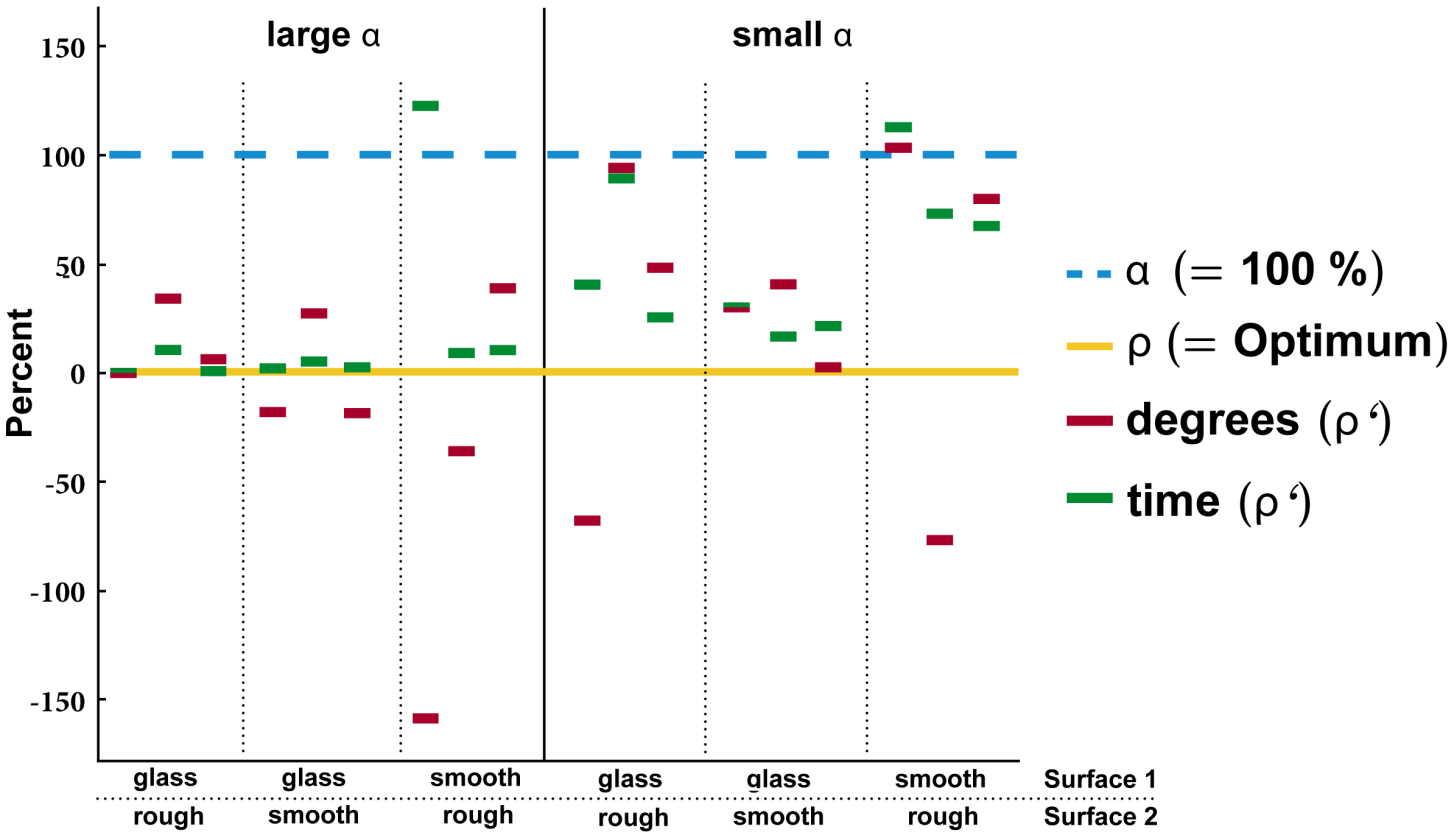
Directions and estimated travel times of ant trails (*ρ’*) relative to the prediction (*ρ*, 0%) and the direct path (*α*, 100%). *W. auropunctata* foragers realized time budgets closer to the optimum *ρ* than to the direct path *α* in 13 out of 18 trials. When moving across glass-felt, travel times were closer to *ρ* than for other surface combinations, and the ants performed better at the larger *α* angle.

The speed at which the ants walk over the surfaces is a crucial determinant of the predicted angle *ρ*. We assessed the walking speed of randomly chosen individuals by filming the final established trail of each colony in each trial. In each of 18 test and six control trials, we traced 40 ants per substrate (one substrate per control trial and two per test trial) and direction (food or nest), yielding a total of 3360 measurements. Along with each individual speed measurement, we estimated trail density by counting the ants on the trail when we traced the walking individuals. VirtualDub (virtualdub.org) was used to calculate the time from start to stop frame (15/s) over a straight 4 cm distance on the ant trail We did not measure the exact distance each ant traveled, which presumably shows minor variation due to differences in head-on encounters of foragers of opposing directions. Instead, we were interested in the mean time a standard ant from each of the three colonies takes to cross a given distance on a specific substrate. We performed a generalized linear model (GLM) in R on these speed data and explored which factors affected trial-specific speed. It is not unlikely that the felt materials for each replicate differed due to fabrication and handling (i.e. a few more erect felt fibers in their path will certainly slow down these minute ants, see Fig. 3) and that colonies furthermore varied due to foraging intensity. Thus, we used speed as response variable and colony, surface (glass, smooth and rough felt), direction of the trail, trail density (number of workers) and angle *α* (30° and 60°) as explanatory variables to test which of them affected speed. As most explanatory variables had a significant impact on speed (see “Results”), we used specific speed means of each particular trial for the following calculus. Because we were interested in the overall speed of the ants during foraging – which includes both traveling to the food and food transport to the nest – we pooled, per trial and substrate, all 40 inbound and 40 outbound measurements and calculated the mean over those 80 values.

### Calculation of Travel Time and *ρ*

Fermat’s principle predicts the fastest path with angle *ρ* for ants to travel between the bridge and the food, located on different surfaces (Fig. 1). For each trial we modeled the traveling time of the ants using the walking speed *v*_x_ for surface *x* (*x* = 1, 2) and the distance *d_x_* the ants hypothetically travel over each surface, depending on the angle *ρ*.

Given are *α*, *a*_1_, and *a*_2_. Using trigonometry, the following equations can be derived:
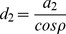
(1)

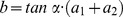
(2)

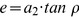
(3)


Because the ratio *a*_1_/*a*_2_ is a crucial determinant of *ρ* and not the actual single segment length, *a*_1_ is replaced by the following term:
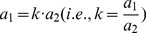
(4)


Combining equations 2 to 4, that part of the predicted path which lies in substrate 1 (*d*_1_) can be calculated as:
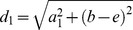


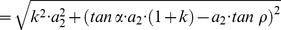


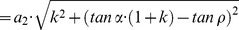
(5)


Using Eq. 1 and 5 we set up a travel time function in dependence from *ρ*:
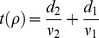


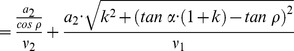


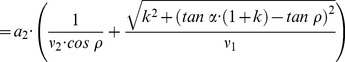



The *ρ* value belonging to the time minimum was approximated to two decimals by calculating t(*ρ*) for 0°<*ρ*<*α* in 0.01° steps using a custom-made Excel sheet (REFRANTZ 1.0, see supporting File S1). The angle *ρ* depends on the differences in speed on the two substrates, thus the larger this difference, the smaller *ρ* will be. Importantly, because the colonies varied in their individual speeds the predicted angle *ρ* varied accordingly among colonies.

### Statistical Analysis of Angle Measurements

We tested two hypotheses: (H1) The ants establish paths following Fermat’s principle, i.e., *ρ′ = ρ*; (H2) the ants follow the direct path, i.e., *ρ′ = α*. Each hypothesis was treated as null hypothesis in a two-tailed sign test [14]; input values were the signs (+/−) of angle differences (paired by trial, i.e., n = 18; H1: *ρ′ – ρ*; H2: *α – ρ′*).

## Results

We found that colonies varied in their speeds (*df = *2, *F* = 38.44, *p*<0.001), that the materials affected speed differently (*df* = 2, *F* = 2297.81, *p*<0.001) (mean ± SD over all colonies in mm/s; polyethylene glass: 4.89±0.51; smooth felt: 2.97±0.91; rough felt: 1.73±0.73), that the ants walked faster from the food to the nest than outbound (*df* = 1, *F* = 47.46, *p*<0.001) and that trail density was negatively correlated with speed (*df* = 1, *F* = 968.41, *p*<0.001). As expected, the angle at which the food was positioned did not affect speed (*df* = 1, *F* = 1.94, *p* = 0.164).

**Figure 4:**
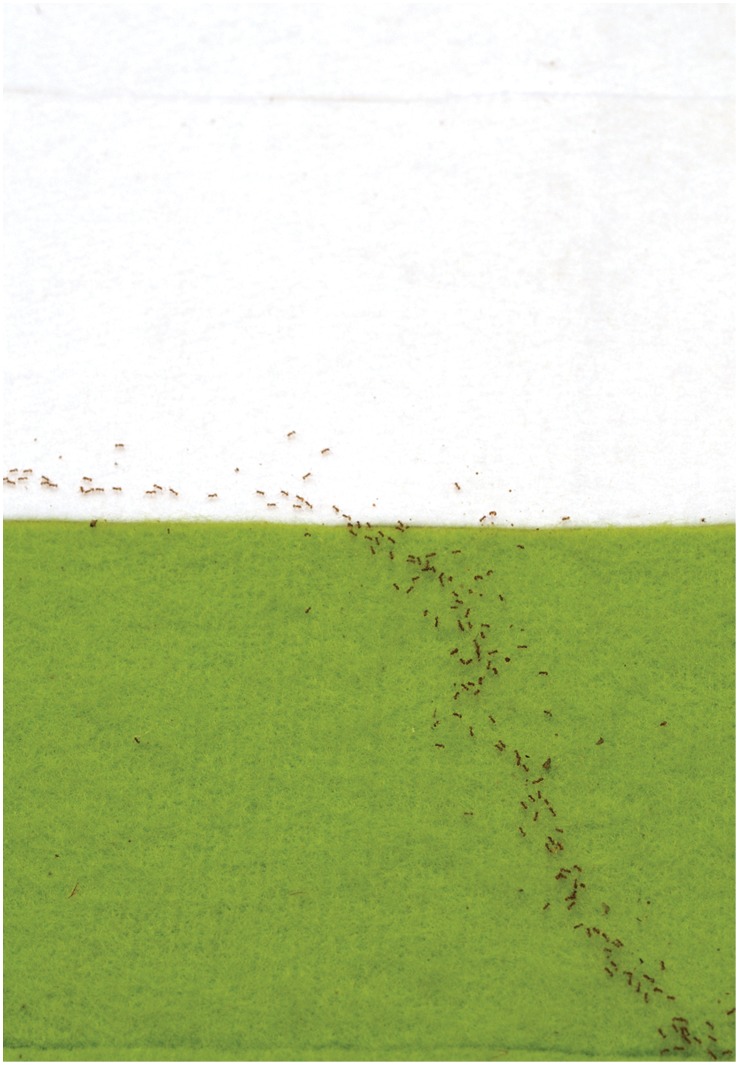
“Refracted” trail of *W. auropunctata* workers at the medium border between smooth (white) and rough (green) felt. The position of the food is on the rough felt. Note that the density of workers on the rough felt is higher than on the smooth felt because travel speed is lower. In addition it appears, although not very obvious, as if the ants on the rough felt ‘float’ on top of the felt hairs, indicating the difficulty of walking on this substrate. Photograph kindly provided by Simon Tragust.

Our test trials showed that *W. auropunctata* ants indeed established trails that significantly deviated from the most direct path (sign test: n+ = 17, n- = 1, *p* = 0.0001). In contrast, the realized paths were overall not different from the predicted angle *ρ* (sign test: n+ = 12, n- = 6, *p* = 0.2379; Fig. 2, Fig. 4). In the controls the trails followed an almost straight line, with the exception of one colony that formed very diffuse trails and for which only one angle could be measured.

Taken together, the experiment affected angle stronger than travel time. The ants realized trails with an angle *ρ′* that differed from the most direct path but did not differ from the fastest path with a predicted *ρ*.

## Discussion

Our study documents that Fermat’s least-time principle, a general rule well known from optics, also applies in biological path-optimization processes. Rather than laying a trail that directly connects nest and food, *Wasmannia* ants reacted to differences in the roughness of the ground and consequently to their running speed by using “refracted” trails that facilitated reduction of travel time. We only detected few cases where the ants perfectly reached the optimum, suggesting that constraints other than the need to optimize time also influence trail formation.

Although “food position” was not significantly correlated with the chosen path a closer inspection revealed that there was a trend that at the larger *α* value (60°) the realized angles deviated more from the optimum than their corresponding realized travel times (Fig. 3). This indicates that with regard to travel time the realized trails were even efficient when angles with deviations from the optimum were established. At the smaller *α* value (i.e. shorter distance) both time and angle showed more variance than at the large *α* value (Fig. 3), suggesting that distance (and constraints of volatile trail pheromones, see below) also play a role in trail formation.

The effect was stronger on angle than on travel time, i.e. drastic deviations from *ρ* do not necessarily result in similar drastic changes in travel time (Fig. 3). This may be a result of the small differences in substrate-specific travel times and the inaccuracy of predicting trail formation from a comparably small subset of foraging ants. Overall however, our simple experiment shows that in central-place foraging species it is not simply the distance, but more precisely the time and thus energy spent that shapes travel routes, even when the benefits are presumably low.

Mass-recruiting ants have long been known to choose the shortest of several routes to a food source (all other things, including surface properties, being equal), and recent studies show that they do so even in complex mazes [9]. In addition, several observations suggest that rather than minimizing travel distance they may optimize travel time or net energy gain. For example, *Pogonomyrmex* harvester ants preferentially utilize low vegetation cover pathways [15], and foragers of the wood ant *Formica rufa* prefer horizontal over vertical bridges of equal length [16]. Furthermore, repulsive interactions cause *Lasius niger* ants to choose the less crowded of two paths and to consequently also minimize travel time [17], [18].

Alternatively one might argue, when looking at the *ρ′* angles measured in our experiment, that the foragers had an intrinsic tendency to establish angles around 10–35°. However, with the substrates available we could for heterogeneous surface combinations only reasonably set up predictions of 10–40°. For predictions below 10°, substrates even more coarse would be needed. Similarly, for larger predicted angles one would need substrates more similar to each other, with the angle of the direct path (*α*) set large enough to be distinguished properly from the optimal path. The last control trial at 60° shows that the ants readily crossed borders between grounds of equal roughness at a much higher angle even after a long trial period with the exception of one colony. This colony decreased in worker number following one year in the lab, several pre-trials and final trials and simply may have lacked the necessary number of workers to maintain a functional trail.

*Wasmannia* workers thus resemble pedestrians, who apparently also follow Fermat’s principle when forced to cross surfaces with different qualities (e.g., [19], [20]). And exactly like humans [21], they appear to tolerate suboptimal detours if they do not grossly inflate travel time. In most of our trials (except the controls), the ants formed trails that over some distance followed the border between the two substrates on the nest-bound half of the arena, suggesting that they prefer to move along edges. Landmarks facilitate orientation [22], a typical ant behavior that can be observed for example on the edges of sidewalks worldwide. This in turn may positively affect the efficiency of pheromone deposition in that lower amounts are needed when pheromone trails are combined with physical cues. Even though ants under natural conditions will forage on a variety of grounds with different resistance [23], foraging, recruitment and trail formation in the field appear to be affected by so many additional factors, such as predation risk or competition [24], [25], that “refraction” as in our experiment will presumably be detected only under specific conditions.

Like archerfish [26], humans are capable of learning to apply the principle of refraction. While these are examples for individual learning and experience, the formation of a collectively established, “refracted” path in ant foraging is based on the properties of volatile pheromones that evaporate over time [27], [28]. Though we did not study the early dynamics of path-finding, we suggest that the foraging trail will initially be determined by chance and subsequently converge towards the optimum. Quicker variants of the trail will be reinforced preferentially, analogously to ant behavior in classic short-cut experiments [8]. The process of trail optimization thus resembles Manfred Eigen’s concept of self-organization and evolution [29], in that information mutates and becomes subject to selection. Our study shows that this procedure allows ants to efficiently optimize time consumption on foraging trails under artificial conditions. But this study also suggests that there are limits to pheromone orientation. When the distance was shorter the ants performed less well maybe as a result of higher pheromone amounts per area unit. Data on the specific pheromones involved and their evaporation rates on different substrates are needed to fully explore trail formation in the little fire ant.

## Supporting Information

File S1 **RERANTZ V1.0. [refrantz1-0.xls], Excel file for the approximation of the fastest trail** ***ρ*** **.**
(ZIP)
